# Protocol for development of an AI-driven individualized frailty prediction and intervention framework for elderly patients with chronic kidney disease: causal feature learning and knowledge-distillation-based modeling study

**DOI:** 10.1186/s12877-026-07143-0

**Published:** 2026-02-12

**Authors:** Jing Chang, Jing Hu, Ying Cao, Xibin Jia, Luo Wang, Bo Liu, Shaowu Xu, Qing Ji, Meiling Jin, Qiuxia Han, Yuer Liang, Qianmei Sun

**Affiliations:** 1 Department of Internal Medicine, Beijing Chao-yang Hospital, Capital Medical University, Beijing, 100020 China; 2 Department of Nephrology, Beijing Chao-yang Hospital, Capital Medical University, Beijing, 100020 China; 3 Department of General Medicine, Zhangjiawan Community Health Service Center of Tongzhou District, Beijing, 101113 China; 4 College of Computer Science, Beijing University of Technology, Beijing, 100124 China

**Keywords:** Frailty, Chronic kidney disease, Causal learning, Knowledge distillation, Artificial intelligence, Risk stratification

## Abstract

**Background:**

Frailty is a reversible geriatric syndrome marked by diminished physiological reserve and heightened vulnerability. In elderly patients with chronic kidney disease (CKD), frailty accelerates decline and mortality, yet individualized risk stratification and management remain limited by resource-intensive geriatric assessment and incomplete data. Integrating causal learning and model knowledge distillation may enable precision modeling of frailty risk and clinically meaningful decision support.

**Methods:**

This multicenter bidirectional cohort study (ChiCTR2500095133) will recruit 1500 adults aged ≥ 60 years with CKD (KDIGO criteria) from seven Beijing centers. Collected data will include routinely available clinical indices (demographics, medical history, anthropometric measures and laboratory tests) and resource-intensive non-clinical indices derived from comprehensive geriatric assessment (frailty, cognitive, functional, nutritional, and psychological assessments). Frailty will be assessed using the FRAIL scale. Causal feature learning will identify determinants of frailty risk, while a teacher-student knowledge-distillation framework will optimize prediction using routinely available clinical data. Internal and external validation will examine model accuracy, interpretability, and clinical applicability.

**Discussion:**

By combining comprehensive clinical assessment with advanced artificial-intelligence methods, this study aims to develop an explainable, efficient tool for early frailty detection and individualized intervention decision support in elderly CKD. The resulting framework may enhance precision geriatric management, improve functional outcomes, and reduce healthcare burden.

**Trial registration:**

Chinese Clinical Trial Registry: ChiCTR2500095133 (registered 2025-01-02).

**Supplementary Information:**

The online version contains supplementary material available at 10.1186/s12877-026-07143-0.

## Background

With population aging accelerating, CKD has emerged as a global health priority, affecting 8–16% of adults and up to nearly 40% of older adults [1, 2]. Frailty, defined as a state of reduced physiological reserve and resilience, is a frequent and severe CKD comorbidity, significantly worsening disability, hospitalization, and mortality [3–6]. Prevalence studies report frailty in 20–50% of older CKD populations [7], driven by metabolic derangements, inflammation, sarcopenia, and multimorbidity [8–10].

Frailty in CKD is clinically important yet potentially reversible [11–13]. Multicomponent interventions combining nutrition, exercise, and psychosocial support have demonstrated benefit, but personalized decision frameworks remain underdeveloped [14–17]. Current screening tools, such as Fried’s phenotype or the FRAIL scale, offer population-level risk estimation but limited mechanistic insight or individualized guidance [18–21].

Artificial-intelligence (AI) methods now offer novel avenues for precision frailty management [22–25], particularly through causal learning and knowledge distillation. However, in routine nephrology practice, non-clinical indices derived from comprehensive geriatric assessment are often unavailable because of resource and workforce constraints, highlighting the need for scalable modeling strategies that can operate under incomplete data conditions. Causal learning disentangles interdependent disease mechanisms, revealing modifiable determinants rather than mere associations [26–28]. Knowledge distillation enables translation from complex multimodal teacher models to lightweight “student” models deployable in clinical settings [29–31]. Applying these methods to CKD-related frailty may bridge data-driven discovery with explainable, clinically actionable models.

This study aims to integrate geriatric assessment with AI-based causal reasoning to (1) identify key frailty-related factors in elderly CKD, (2) develop predictive and interventional-support models using causal learning and model distillation, and (3) evaluate potential real-world clinical feasibility and applicability of the proposed framework.

## Methods

### Study design

**Fig. 1:**
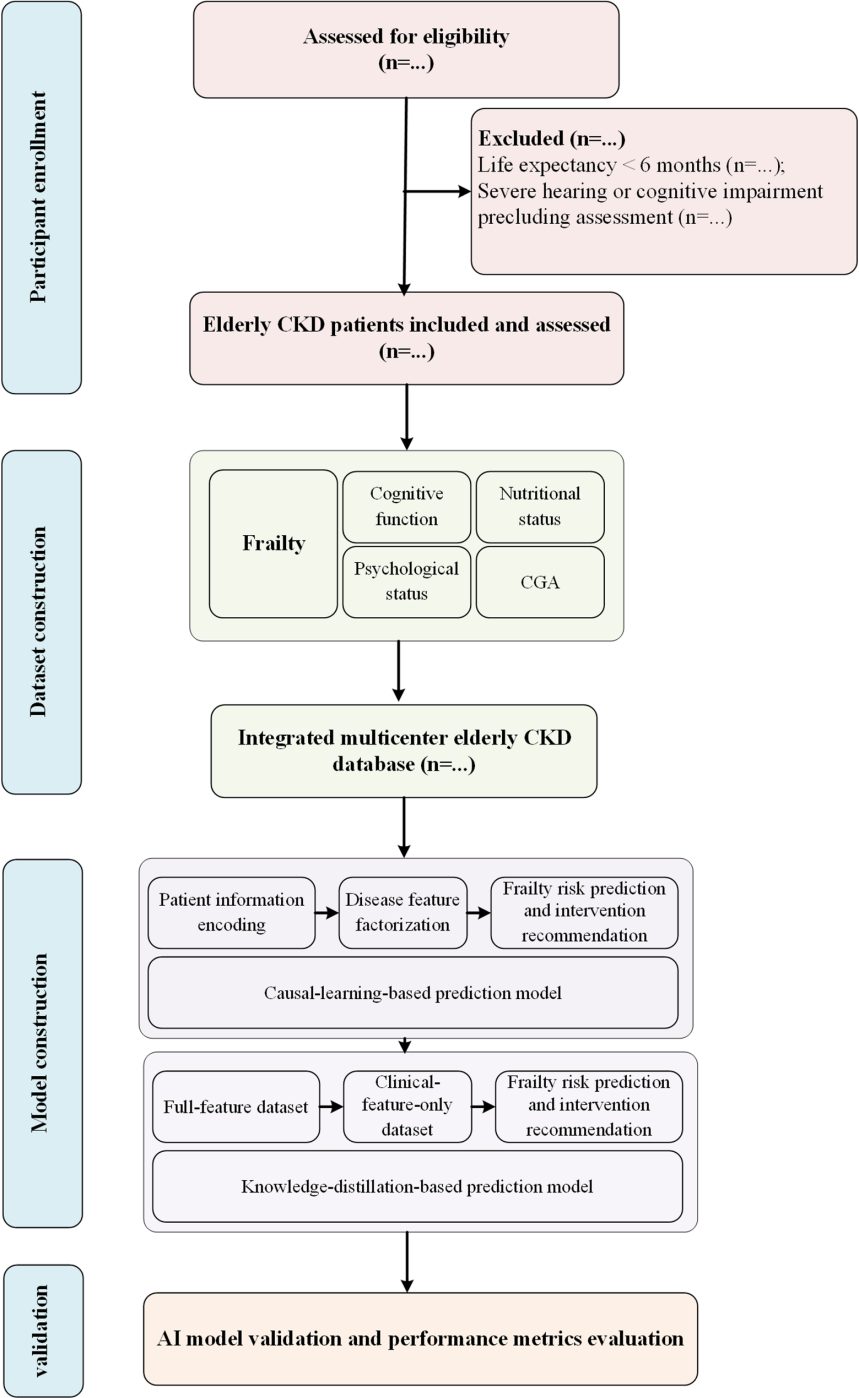
Study flow diagram of the multicenter bidirectional cohort and AI-based frailty modeling framework

A multicenter bidirectional cohort study is being conducted from December 1, 2024, to December 31, 2028 (Fig. 1). Beijing Chaoyang Hospital serves as the coordinating center, with six participating sites across tertiary and community hospitals. Both retrospective data extraction and prospective recruitment will be employed to establish a bidirectional cohort integrating hospital and community populations. Ethical approval has been obtained from the Institutional Review Board of Beijing Chaoyang Hospital (No. 2024-Science-914). All participants will provide written informed consent. The trial adheres to the Declaration of Helsinki and SPIRIT 2023 guidelines.

### Participants

#### Inclusion criteria

Age ≥ 60 years; Diagnosis of CKD per KDIGO 2024 criteria (GFR < 60 mL/min/1.73 m² or structural abnormality > 3 months); Capacity and willingness to provide informed consent.

#### Exclusion criteria 

Life expectancy < 6 months (e.g., advanced malignancy); Severe hearing or cognitive impairment precluding assessment.

### Sample size justification

The target sample size is 1,500 participants, based on both prediction-modeling and prevalence-precision considerations. Assuming a frailty prevalence of 20% [3, 7] and approximately 30 candidate predictors, the events-per-variable (EPV) rule (≥ 10) [32, 33] yields the minimum required sample:

$$\:n=\frac{10\times\:30}{0.20}=\mathrm{1,500}$$

This ensures adequate frailty events (~ 300) for stable model estimation. Additionally, applying the binomial precision formula for a single proportion,

$$\:n=\frac{{z}_{1-\alpha\:/2}^{2}p(1-p)}{{m}^{2}}$$

a total of 1,500 participants provides ± 2% precision (95% CI) around a frailty prevalence of 20%, which is acceptable for multi-center estimation. The chosen size thus satisfies both statistical and practical requirements for developing and externally validating a robust, interpretable prediction model.

### Recruitment and data collection

This is a multicenter, bidirectional cohort study. Eligible participants will be consecutively enrolled from nephrology and internal medicine departments across seven hospitals in Beijing. Retrospective data extraction will be performed only at the coordinating center (Beijing Chaoyang Hospital), while all participating centers will contribute prospective data collection. Pre-admission and admission-related baseline data, including demographic, clinical, and laboratory characteristics, will be retrospectively extracted from the electronic medical records (EMRs) at the coordinating center for eligible historical cases, while baseline standardized assessments will be prospectively performed at hospital admission for newly enrolled participants across all participating centers starting on January 3, 2025. During the prospective data collection phase, each participant will undergo standardized assessments encompassing: structured questionnaires for demographics, comorbidities, physical examinations and anthropometric measurements; laboratory tests including blood and urine biochemistry, inflammatory and metabolic indicators; geriatric assessment scales, frailty, cognition, nutrition, psychological state, physical performance. Collected variables were categorized into two groups: routinely available clinical indices (demographics, medical history, anthropometric measures and laboratory tests) and non-clinical indices, defined as non-routinely acquired geriatric assessment data (frailty scales and cognitive, functional, nutritional, and psychological assessments), which require specialized evaluation and are resource-intensive in routine clinical practice. All data will be entered into a harmonized electronic case-report form (eCRF). The database will be centrally curated at Beijing Chaoyang Hospital, following prespecified data quality control and harmonization procedures described below.

### Multicenter quality assurance and data management plan

To ensure consistency across multiple centers and minimize heterogeneity, our research team implemented standardized procedures for assessment implementation, data collection, and quality control across all participating sites.

Unified Training and Standard Operating Procedures (SOPs): All investigators and research staff underwent centralized training before study initiation. This included detailed instructions on assessment implementation, data collection procedures, outcome assessment criteria and standardized delivery of multicomponent intervention recommendations across sites. Standardized assessment manuals and implementation checklists were distributed to each center, and regular online meetings were conducted to reinforce protocol adherence. A standardized case-report form (CRF) was developed and used uniformly across all sites to ensure consistent data recording.

Data Standardization and Quality Control: We established a multi-level data cleaning and quality control process. First, automated validation rules were embedded in the electronic data capture (EDC) system to check for completeness and logical consistency at the time of data entry. Second, centralized outlier detection and missing-data handling procedures were applied. Third, independent data verification was performed to ensure cross-field consistency and data integrity.

Centralized Data Management and Feedback Loop: A dedicated data management team was responsible for regular monitoring, data cleaning, and feedback to each participating center. Any discrepancies or missing data were promptly communicated to the sites for resolution, ensuring the reliability and completeness of the final dataset.

All procedures were conducted in accordance with the Guidelines for Data Management in Clinical Research, ensuring high-quality data to support subsequent modeling and analysis.

### Measurements

#### Primary outcome

Frailty status in elderly CKD patients is defined using the FRAIL scale (Fatigue, Resistance, Ambulation, Illness, and Weight Loss; total score ≥ 3 indicating frailty). The primary analytical objective is to examine associations between clinical and non-clinical indices and frailty status and to support the development of individualized frailty risk prediction models.

#### Secondary outcomes

Secondary outcomes comprise multidomain geriatric indicators related to frailty and intervention guidance: Cognitive function: Mini-Mental State Examination (MMSE) or Montreal Cognitive Assessment (MoCA); Nutritional status: Mini Nutritional Assessment–Short Form (MNA-SF); Psychological status: anxiety and depression; Comprehensive Geriatric Assessment (CGA) including physical performance (gait speed, grip strength, chair-stand test), functional ability (ADL, IADL).

Variable definitions will follow KDIGO 2024 and AWGS 2019 guidelines to ensure inter-center comparability. The integrated dataset will be used to identify older CKD patients at high risk for frailty and to generate individualized decision-support recommendations for nutritional, psychological, and physical-activity interventions through interpretable AI models.

### Model development and validation

**Fig. 2:**
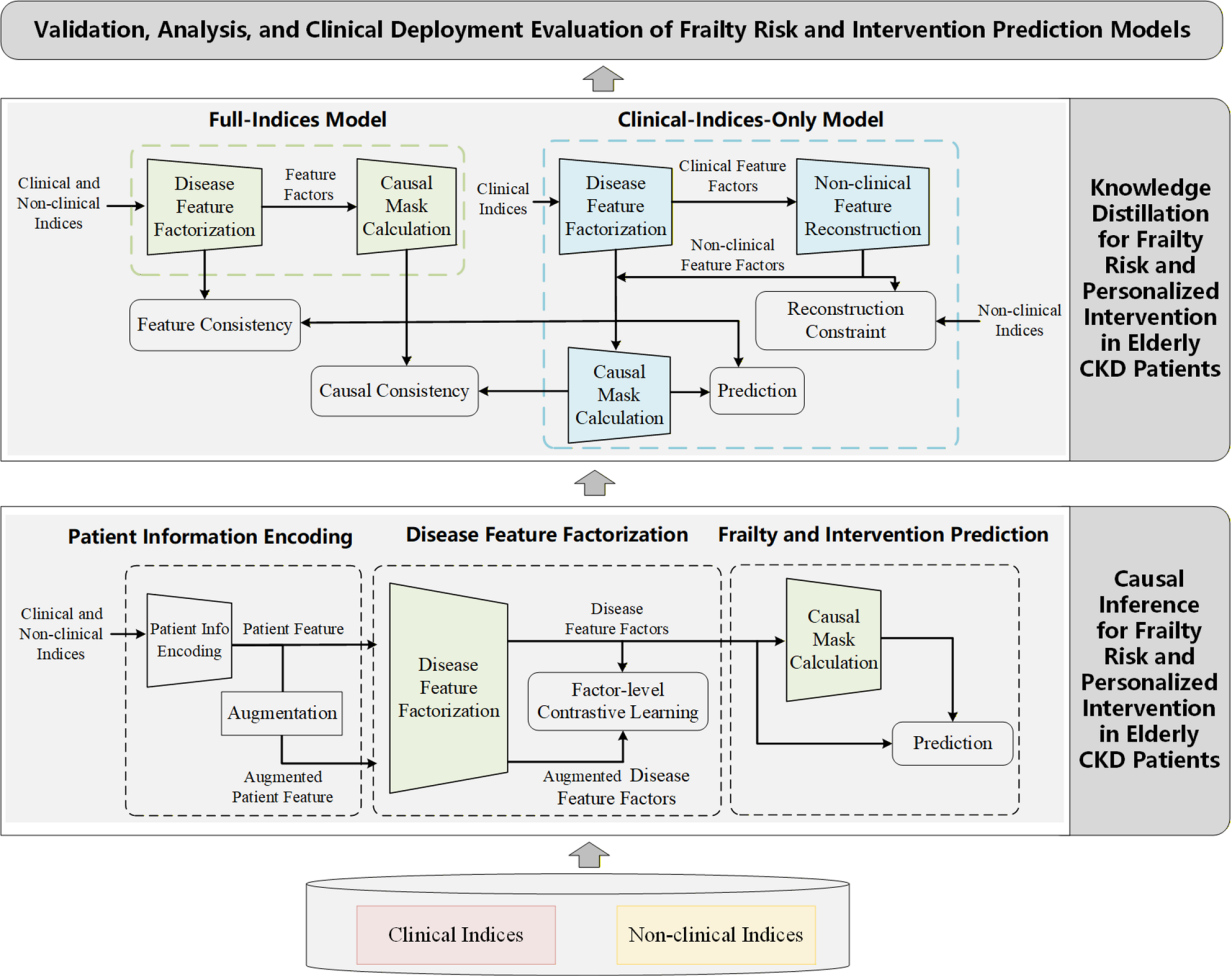
Overview of the proposed causal-learning and knowledge-distillation framework for frailty risk prediction and individualized intervention in elderly patients with CKD. Clinical indices represent routinely available clinical variables (demographics, medical history, anthropometric measures and laboratory tests), while non-clinical indices represent resource-intensive geriatric assessment data (frailty, cognitive, functional, nutritional, and psychological assessments). The full-indices model is trained using both clinical and non-clinical indices, whereas the clinical-indices-only model is optimized through knowledge distillation to enable frailty risk prediction and intervention decision support in data-limited clinical settings

An overview of the proposed AI modeling framework, including causal learning, knowledge distillation, and clinical deployment workflow, is illustrated in Fig. 2.

### Causal-learning-based frailty modeling

Patient-level clinical indices and non-clinical indices will first be processed using a structured modeling pipeline, as illustrated in Fig. 2. Raw input variables will be transformed into standardized feature representations through the patient information encoding module. These encoded features will then be input into the disease feature factorization module to generate latent disease-related feature factors. The extracted feature factors will subsequently be used for causal structure estimation.

Based on the learned feature relationships, a causal mask will be constructed to represent the dependency constraints among variables. This causal mask will be integrated into the downstream prediction network to guide feature interaction and information flow during model inference. Finally, the masked feature representations will be used as inputs to the prediction module to generate frailty risk estimates and intervention decision-support outputs for elderly patients with CKD.

### Knowledge-distillation-based model optimization

A teacher–student learning framework will be implemented. The teacher model is trained using full indices including both clinical and non-clinical indices. The student model, restricted to routinely available clinical indices only, is trained by minimizing the distillation loss between teacher and student outputs, enabling the student model to learn compressed representations capturing information patterns related to geriatric assessment and frailty evaluation from routinely available clinical data. This strategy allows simplified deployment of the prediction model in routine clinical environments with limited data availability.

### Clinical workflow integration and deployment pathway

After model training and validation, the optimized student model will be integrated into an electronic medical record (EMR)–linked clinical decision-support interface. Routinely available clinical indices will be automatically extracted as model inputs. The system will generate individualized frailty risk scores and intervention suggestions, which will be presented to nephrologists and geriatricians through a structured dashboard. Model outputs will serve as assistive decision-support information, while final clinical decisions will remain under physician supervision, ensuring a human-in-the-loop implementation strategy.

### Validation and evaluation

Internal validation, 70% of data used for model training and 30% for testing within the Chaoyang Hospital dataset. External validation, independent testing using data from six partner centers. Performance metrics, AUC, calibration curve, Brier score, and decision-curve analysis. Model explainability, assessed using SHAP-based feature attribution and causal-effect visualization to ensure interpretability and clinical credibility.

### Outcomes and analysis

This study is non-interventional and does not include follow-up endpoints. The analyses will focus on: Descriptive analysis of demographic and clinical characteristics, and frailty prevalence; Causal learning to determine key factors contributing to frailty in elderly CKD; Predictive model construction and validation using machine-learning and causal frameworks; Simulation-based recommendation system, producing interpretable suggestions for targeted nutritional, psychological, and physical interventions.

### Statistical plan

Continuous variables will be presented as mean ± SD or median (IQR); categorical variables as frequency (%). Group comparisons will use Student’s *t* test, χ² test, or Mann–Whitney *U* test as appropriate. Causal effects will be estimated using do-calculus, counterfactual simulation, and feature-importance mapping. All analyses will be performed using R 4.3 and Python 3.11.

### Missing data

Multiple imputation by chained equations (MICE) will be applied under the missing-at-random assumption. Sensitivity analyses will verify robustness.

### Ethics and data security

De-identified data will be stored in secure servers with role-based access. Any protocol amendment will be reported to the central ethics committee and the trial registry.

## Discussion

Frailty in elderly CKD represents a dynamic, modifiable continuum rather than a fixed state [11–13, 34, 35]. Traditional assessments fail to capture individualized causal pathways linking renal dysfunction, inflammation, and functional decline [18–21]. By employing causal learning, this study aims to move beyond purely associative modeling toward improved mechanistic interpretability and clinically meaningful risk stratification [26–28], while knowledge distillation is expected to enhance scalability and applicability of the prediction framework in routine clinical settings with heterogeneous data availability [29–31].

This framework is designed to address real-world heterogeneity in frailty assessment availability across different healthcare settings. Comprehensive geriatric assessment is resource-intensive and not uniformly implemented across hospitals with different service capacities; consequently, many older patients with CKD lack complete geriatric assessment data and are often evaluated using routine clinical indicators only. To address this practical constraint, the proposed teacher–student framework is designed to extend frailty screening and intervention decision-support to data-limited clinical environments. The teacher model leverages comprehensive multimodal data when available, whereas the student model is optimized to operate using routinely collected laboratory and clinical variables. This design enables frailty screening and risk stratification under incomplete data conditions and supports broader applicability across healthcare settings with variable resource availability. In addition, the multicenter study design facilitates model development across heterogeneous CKD populations with different disease severity and comorbidity profiles, further supporting adaptability in diverse clinical environments.

The expected outcomes include the development of an interpretable AI-assisted frailty prediction framework, establishment of a multicenter CKD–frailty database, and provision of quantitative decision-support outputs to assist individualized risk stratification and care planning. In clinical practice, the proposed framework is intended to support frailty risk stratification and individualized care planning rather than replace clinical judgment. Model-generated frailty risk scores and interpretable feature contributions may assist clinicians in identifying high-risk patients who could benefit from comprehensive geriatric assessment and targeted interventions, while facilitating routine monitoring of lower-risk individuals in primary care settings. At the population level, standardized risk outputs derived from multicenter data may contribute to evidence generation for defining frailty risk thresholds and stratified management pathways, thereby providing supportive data for future clinical pathway optimization and guideline refinement.

Several limitations should also be acknowledged. First, model performance may be influenced by temporal changes in patient characteristics and treatment patterns, potentially leading to model drift. To minimize this impact, continuous data updating and periodic model recalibration will be implemented within the bidirectional cohort framework. Second, although knowledge-distillation techniques enable prediction using laboratory-only inputs, the inferred representations of clinician-rated frailty and geriatric assessment information derived from laboratory features require further validation. This limitation will be addressed through prospective data collection and external validation across broader and more diverse patient populations. Third, residual heterogeneity across participating centers may persist due to variations in clinical practice patterns and healthcare infrastructure. This will be mitigated through standardized assessment and implementation protocols, centralized quality control procedures, and harmonized data management strategies implemented across all study sites.

Ultimately, this project aims to integrate advanced computational modeling into everyday geriatric nephrology workflows, providing clinicians with supportive, patient-specific risk information while maintaining physician oversight and final clinical decision authority, thereby supporting safe and responsible integration into routine geriatric nephrology workflows.

### Trial status

Recruitment commenced on January 3, 2025, with completion anticipated by December 31, 2027. Final analyses are anticipated by mid-2028.

## Supplementary Information

Supplementary Material 1.

## Data Availability

Data will be available from the corresponding author upon reasonable request after study completion.

## Abbreviations

CKD: Chronic Kidney Disease
FRAIL: Fatigue, Resistance, Ambulation, Illness, Loss of Weight
MNA: SF—Mini Nutritional Assessment Short Form
MMSE: Mini Mental State Examination
MoCA: Montreal Cognitive Assessment
GDS: Geriatric Depression Scale
KDIGO: Kidney Disease: Improving Global Outcomes
AI: Artificial Intelligence

## References

[1] Chen TK, Knicely DH, Grams ME. Chronic kidney disease diagnosis and management. JAMA. 2019;322:1294–304. 10.1001/jama.2019.14745. 31573641 10.1001/jama.2019.14745 PMC7015670

[2] Merchant AA, Ling E. An approach to treating older adults with chronic kidney disease. CMAJ Can Med Assoc J. 2023;195:E612–8. 10.1503/cmaj.221427. 37127307 10.1503/cmaj.221427 PMC10151089

[3] Fried LP, Tangen CM, Walston J, Newman AB, Hirsch C, Gottdiener J, et al. Frailty in older adults: evidence for a phenotype. J Gerontol Biol Sci Med Sci. 2001;56:M146–156. 10.1093/gerona/56.3.m146. 10.1093/gerona/56.3.m146 11253156

[4] Dent E, Martin FC, Bergman H, Woo J, Romero-Ortuno R, Walston JD. Management of frailty: opportunities, challenges, and future directions. Lancet Lond Engl. 2019;394:1376–86. 10.1016/S0140-6736(19)31785-4. 10.1016/S0140-6736(19)31785-4 31609229

[5] Taylor JA, Greenhaff PL, Bartlett DB, Jackson TA, Duggal NA, Lord JM. Multisystem physiological perspective of human frailty and its modulation by physical activity. Physiol Rev. 2022;103:1137. 10.1152/physrev.00037.2021. 36239451 10.1152/physrev.00037.2021 PMC9886361

[6] Wilkinson TJ, Miksza J, Zaccardi F, Lawson C, Nixon AC, Young HML, et al. Associations between frailty trajectories and cardiovascular, renal, and mortality outcomes in chronic kidney disease. J Cachexia Sarcopenia Muscle. 2022;13:2426–35. 10.1002/jcsm.13047. 35851589 10.1002/jcsm.13047 PMC9530530

[7] Li B-H, Sang N, Zhang M-Y, Liu Z-R, Fang R-X, Liu W-J, et al. The prevalence and influencing factors of frailty in patients with chronic kidney disease: a systematic review and meta-analysis. Int Urol Nephrol. 2024;56:767–79. 10.1007/s11255-023-03739-2. 37578673 10.1007/s11255-023-03739-2

[8] Chan GC-K, Kalantar-Zadeh K, Ng JK-C, Tian N, Burns A, Chow K-M, et al. Frailty in patients on Dialysis. Kidney Int. 2024;106:35–49. 10.1016/j.kint.2024.02.026. 38705274 10.1016/j.kint.2024.02.026

[9] Arosio B, Calvani R, Ferri E, Coelho-Junior HJ, Carandina A, Campanelli F, et al. Sarcopenia and cognitive decline in older adults: targeting the Muscle–Brain axis. Nutrients. 2023;15:1853. 10.3390/nu15081853. 37111070 10.3390/nu15081853 PMC10142447

[10] Vetrano DL, Palmer K, Marengoni A, Marzetti E, Lattanzio F, Roller-Wirnsberger R, et al. Frailty and multimorbidity: A systematic review and Meta-analysis. J Gerontol Biol Sci Med Sci. 2019;74:659–66. 10.1093/gerona/gly110. 10.1093/gerona/gly110 29726918

[11] Sun X, Wang C, Zheng R, Liu Z, Song W, Du X et al. Frailty transitions and risk of chronic kidney disease: insights from the China health and retirement longitudinal study. Ren Fail. 47:2478483. 10.1080/0886022X.2025.2478483. 10.1080/0886022X.2025.2478483 PMC11921158 40101286

[12] Nixon AC, Bampouras TM, Pendleton N, Woywodt A, Mitra S, Dhaygude A. Frailty and chronic kidney disease: current evidence and continuing uncertainties. Clin Kidney J. 2018;11:236–45. 10.1093/ckj/sfx134. 29644065 10.1093/ckj/sfx134 PMC5888002

[13] Travers J, Romero-Ortuno R, Langan J, MacNamara F, McCormack D, McDermott C, et al. Building resilience and reversing frailty: a randomised controlled trial of a primary care intervention for older adults. Age Ageing. 2023;52:afad012. 10.1093/ageing/afad012. 36849160 10.1093/ageing/afad012

[14] Ng TP, Feng L, Nyunt MSZ, Feng L, Niti M, Tan BY, et al. Nutritional, Physical, Cognitive, and combination interventions and frailty reversal among older adults: A randomized controlled trial. Am J Med. 2015;128:1225–e12361. 10.1016/j.amjmed.2015.06.017. 26159634 10.1016/j.amjmed.2015.06.017

[15] Hsieh T-J, Su S-C, Chen C-W, Kang Y-W, Hu M-H, Hsu L-L, et al. Individualized home-based exercise and nutrition interventions improve frailty in older adults: a randomized controlled trial. Int J Behav Nutr Phys Act. 2019;16:119. 10.1186/s12966-019-0855-9. 31791364 10.1186/s12966-019-0855-9 PMC6889427

[16] Nari F, Jang BN, Youn HM, Jeong W, Jang S-I, Park E-C. Frailty transitions and cognitive function among South Korean older adults. Sci Rep. 2021;11:10658. 10.1038/s41598-021-90125-6. 34017031 10.1038/s41598-021-90125-6 PMC8138002

[17] Sirikul W, Buawangpong N, Pinyopornpanish K, Siviroj P. Impact of multicomponent exercise and nutritional supplement interventions for improving physical frailty in community-dwelling older adults: a systematic review and meta-analysis. BMC Geriatr. 2024;24:958. 10.1186/s12877-024-05551-8. 39558234 10.1186/s12877-024-05551-8 PMC11571505

[18] Kennard AL, Glasgow NJ, Rainsford SE, Talaulikar GS. Narrative review: clinical implications and assessment of frailty in patients with advanced CKD. Kidney Int Rep. 2023;9:791–806. 10.1016/j.ekir.2023.12.022. 38765572 10.1016/j.ekir.2023.12.022 PMC11101734

[19] Kennard AL, Rainsford S, Glasgow NJ, Talaulikar GS. Use of frailty assessment instruments in nephrology populations: a scoping review. BMC Geriatr. 2023;23:449. 10.1186/s12877-023-04101-y. 37479978 10.1186/s12877-023-04101-y PMC10360289

[20] Puri A, Lloyd AM, Bello AK, Tonelli M, Campbell SM, Tennankore K, et al. Frailty assessment tools in chronic kidney disease: A systematic review and Meta-analysis. Kidney Med. 2025;7:100960. 10.1016/j.xkme.2024.100960. 39980935 10.1016/j.xkme.2024.100960 PMC11841092

[21] Fierro-Marrero J, Reina-Varona Á, Paris-Alemany A, La Touche R. Frailty in geriatrics: A critical review with content analysis of Instruments, overlapping Constructs, and challenges in diagnosis and prognostic precision. J Clin Med. 2025;14:1808. 10.3390/jcm14061808. 40142616 10.3390/jcm14061808 PMC11943423

[22] Grobe N, Scheiber J, Zhang H, Garbe C, Wang X. Omics and artificial intelligence in kidney diseases. Adv Kidney Dis Health. 2023;30:47–52. 10.1053/j.akdh.2022.11.005. 36723282 10.1053/j.akdh.2022.11.005

[23] Kee XLJ, Sng GGR, Lim DYZ, Tung JYM, Abdullah HR, Chowdury AR. Use of a large Language model with instruction-tuning for reliable clinical frailty scoring. J Am Geriatr Soc. 2024;72:3849–54. 10.1111/jgs.19114. 39105505 10.1111/jgs.19114

[24] Yamashita M, Kamiya K, Hotta K, Kubota A, Sato K, Maekawa E et al. Artificial intelligence (AI)-Driven frailty prediction using electronic health records in hospitalized patients with cardiovascular disease. Circ Rep 6:495–504. 10.1253/circrep.CR-24-0112. 10.1253/circrep.CR-24-0112 PMC11541179 39525301

[25] Li T, Li X, Xu H, Wang Y, Ren J, Jing S, et al. Machine learning approaches for predicting frailty base on multimorbidities in US adults using NHANES data (1999–2018). Comput Methods Programs Biomed Update. 2024;6:100164. 10.1016/j.cmpbup.2024.100164.

[26] Prosperi M, Guo Y, Sperrin M, Koopman JS, Min JS, He X, et al. Causal inference and counterfactual prediction in machine learning for actionable healthcare. Nat Mach Intell. 2020;2:369–75. 10.1038/s42256-020-0197-y.

[27] Olier I, Zhan Y, Liang X, Volovici V. Causal inference and observational data. BMC Med Res Methodol. 2023;23:227. 10.1186/s12874-023-02058-5. 37821812 10.1186/s12874-023-02058-5 PMC10566026

[28] Schölkopf B, Locatello F, Bauer S, Ke NR, Kalchbrenner N, Goyal A et al. Toward Causal Representation Learning. Proc IEEE. 2021;109:612–34. 10.1109/JPROC.2021.3058954.

[29] Hinton G, Vinyals O, Dean J. Distilling the knowledge in a neural network. 2015. 10.48550/arXiv.1503.02531.

[30] Guerra-Manzanares A, Shamout FE. MIND: Modality-Informed knowledge distillation framework for multimodal clinical prediction tasks. 2025. 10.48550/arXiv.2502.01158

[31] Wu M, Goodman N et al. (2020). Multimodal Learning with Incomplete Modalities by Knowledge Distillation. In Proceedings of the 26th ACM SIGKDD International Conference on Knowledge Discovery & Data Mining (pp. 2038–2048). 10.1145/3394486.3403234.

[32] Peduzzi P, Concato J, Kemper E, Holford TR, Feinstein AR. A simulation study of the number of events per variable in logistic regression analysis. J Clin Epidemiol. 1996;49:1373–9. 10.1016/s0895-4356(96)00236-3. 8970487 10.1016/s0895-4356(96)00236-3

[33] van Smeden M, Moons KG, de Groot JA, Collins GS, Altman DG, Eijkemans MJ, et al. Sample size for binary logistic prediction models: beyond events per variable criteria. Stat Methods Med Res. 2019;28:2455–74. 10.1177/0962280218784726. 29966490 10.1177/0962280218784726 PMC6710621

[34] Stolz E, Mayerl H, Freidl W. Fluctuations in frailty among older adults. Age Ageing. 2019;48:547–52. 10.1093/ageing/afz040. 31028381 10.1093/ageing/afz040

[35] Cohen CI, Benyaminov R, Rahman M, Ngu D, Reinhardt M. Frailty: A multidimensional biopsychosocial syndrome. Med Clin North Am. 2023;107:183–97. 10.1016/j.mcna.2022.04.006. 36402498 10.1016/j.mcna.2022.04.006

